# Intraosseous Schwannoma (Neurilemmoma) of the Cervical Spine

**DOI:** 10.1155/S1357714X01000196

**Published:** 2001-06

**Authors:** H. W. Bart Schreuder, René P. H. Veth, Maciej Pruszczynski, J. Albert M. Lemmens, Erik W. van Laarhoven

**Affiliations:** 1 800 Department of Orthopaedics University Hospital Nijmegen P.O. Box 9101 Nijmegen 6500 HB The Netherlands; 2 Department of Pathology University Hospital Nijmegen Nijmegen The Netherlands; 3 Department of Radiology University Hospital Nijmegen Nijmegen The Netherlands; 4 Department of Orthopaedics Tilburg The Netherlands; 5 Twee Steden Hospital Tilburg The Netherlands

## Abstract

*Purpose:* To report on an extremely rare tumour located in the cervical spine, its treatment and result. Review of the literature.

*Patient:* Case report of a 38-year-old woman with an intraosseous schwannoma of the cervical spine.

*Results:* After local curettage no evidence for local recurrence at long-term follow-up.


*Sarcoma* (2001) **5**, 101–103

**CASE REPORT**

### Intraosseous schwannoma (neurilemmoma) of the cervical spine

H.W. BART SCHREUDER[1], RENÉ P.H. VETH[2], MACIEJ PRUSZCZYNSKI[3], J. ALBERT M. LEMMENS[4] & ERIK W. VAN LAARHOVEN[5]

*Departments of [1]Orthopaedics, [2]Pathology and [3]Radiology, University Hospital Nijmegen, Nijmegen, The Netherlands, and [4]Department of Orthopaedics, [5]Twee Steden Hospital, Tilburg, The Netherlands*

**Abstract**
*Purpose:* To report on an extremely rare tumour located in the cervical spine, its treatment and result. Review of the literature.
*Patient:* Case report of a 38-year-old woman with an intraosseous schwannoma of the cervical spine.
*Results:* After local curettage no evidence for local recurrence at long-term follow-up.

### Introduction

Schwannoma (neurilemmoma) is a benign soft tissue tumour which arises mainly in association with sensory nerves. An intraosseous location is extremely rare and may occur by three mechanisms: by an extraosseous tumour and secondary erosion of bone, by a tumour arising within the nutrient canal which grows in a dumbbell-shaped configuration, producing enlargement of the canal and finally by a tumour arising centrally within the bone.[1]

We describe a case of a cervical intraosseous schwannoma, its treatment and outcome.

### Case report

A 38-year-old female presented with pain in the neck for several months and some dysphagia. The pain had increased gradually, with some paraesthesia to the right scapula. There was no history of trauma and she was otherwise completely healthy.

On examination the range of motion of the cervical column was normal, but flexion was painful. Neurological examination revealed no abnormalities and there were no signs of von Recklinghausen's disease.

The conventional radiograph of the cervical spine showed a missing contour of the ventral part of C6 with destruction of the anterior cortex of the vertebra (Fig. 1A). MR showed a mass originating from the vertebra with displacement of the ventral soft-tissue structures but without signs of local invasive or aggressive growth. In both T1- and T2-weighted images the signal-intensity was high, indicating fatty as well as tissue with high fluid content (Fig. 1B and C). There was no sign of oedema in surrounding tissues.

A technetium nuclear bone scan showed only a moderate local uptake, with no distant abnormalities. The chest X-ray was normal.

Overall the lesion was evaluated as benign and the differential diagnosis included aneurysmal bone cyst with fracture of the anterior wall of the corpus, eosinophilic granuloma of bone, giant cell tumour and schwannoma. Malignant alternatives were metastasis of carcinoma, (solitary) myeloma, chordoma or a sarcoma. Because of the location of the tumour it was decided to perform a one-stage procedure, including a biopsy, a frozen section and, if possible, definitive treatment.

At surgery, through an anterior left-sided longitudinal approach, a well capsulated mass of 3 by 2 cm was found, which seemed to extrude from the C6 vertebral body, passing in front of the C7 vertebral body. Without contaminating the surrounding tissues an incision biopsy was taken for frozen sectioning and the diagnosis schwannoma without any malignant features was made. The extruding mass was excised sharply at the cortical edges of the vertebral body C6. The remaining tumour inside the vertebral body was carefully removed using curettes. The entire wound was irrigated with sodium hypochloride to prevent seeding of tumour cells. Effectively a subtotal corporectomy with removal of the intervertebral disc was performed and a C5–C7 spondylodesis was done using a tricortical bone plug taken from the iliac wing.

Correspondence to: H.W.B. Schreuder, 800 Department of Orthopaedics, University Hospital Nijmegen, P.O. Box 9101, 6500 HB Nijmegen, The Netherlands. Tel.: +31-24-3613918; fax: +31-24-3540230; e-mail: b.schreuder@orthp.azn.nl

1357–714X print/1369–1643 online/01/020101-03 © 2001 Taylor & Francis Ltd
DOI: 10.1080/13577140120048610

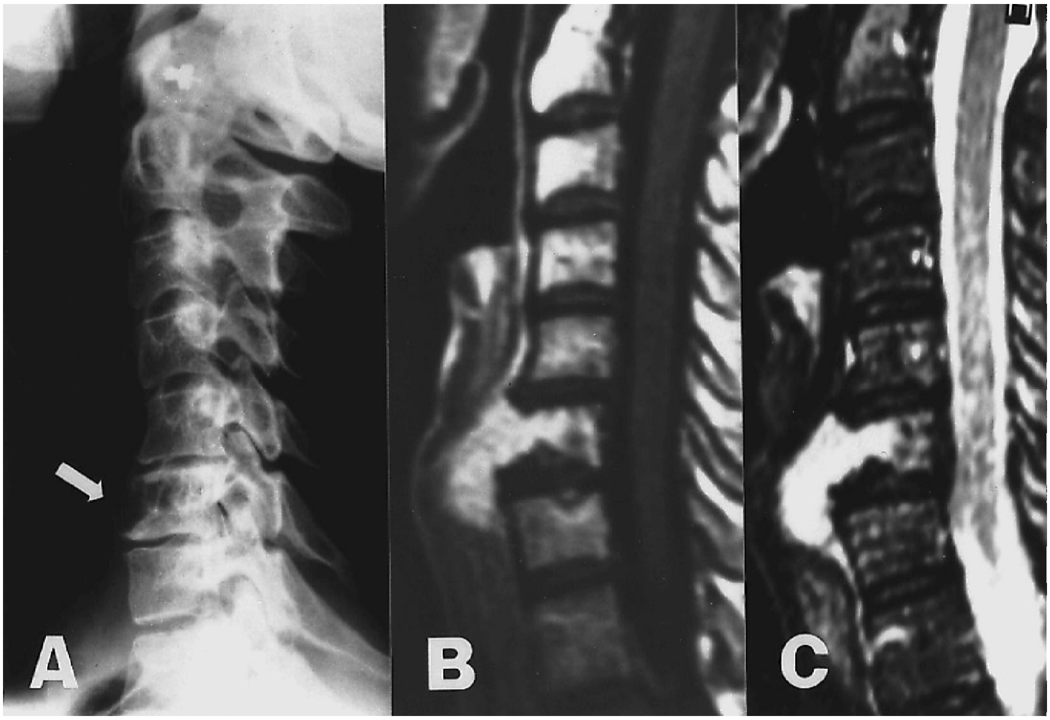

**Fig. 1.** *Lateral routine radiograph of a 38-year-old patient revealing a lytic lesion of the body of C6 with erosion of the anterior cortex (A). MRI ((B) T1; (C) T2) showing a well-demarcated lesion seemingly originating in corpus C6 and protruding anteriorly elevating the spinal anterior ligament.*

Anterior stabilisation was done with a low profile plate and screws (Fig. 2A). The definite histology confirmed the diagnosis of benign schwannoma. The patient recovered quickly and was treated with an orthosis for 3 months. Oncological follow-up consisted of routine radiographs and MRI every 6 months. The conventional radiograph of the cervical spine after 4 years shows a remodelling of the spondylodesis (Fig. 2B). The T1- and T2-weighted MR images do not show any abnormalities, except for some slightly disturbing artifacts originating from the titanium plate (Fig. 2C and D).

### Discussion

Intraosseous schwannoma is an extremely rare entity and the majority of cases reported are located in the mandible and sacrum.[1–5] Clinical onset is usually in patients in their third or fourth decade of life with no sex predilection.[1,6] Since this benign lesion enlarges slowly, the history of the patient may be considerably long and most cases present with pain due to a mass next to other symptoms depending on the location.[1,4]

The radiological features of intraosseous schwannoma consist of a lytic defect with cortical erosion without periosteal new bone formation and absence of central calcification or ossification.[1,4,5,7] Further investigation with MRI and or CT will help to establish the extent of the lesion, its benign growth pattern and differential diagnosis.

Lesions occurring in the vertebral column have been reported in the literature,[8,9] but we are aware of only two case reports reported in the literature of intraosseous schwannoma located in the cervical spine. Polkey described a 34-year-old woman with an expanding lesion of the bodies of C6 and C7 vertebrae causing local neurological signs and spastic paraparesis. Six weeks after dorsal fusion of C5–T1 a complete removal of a well-demarcated tumour was performed in a piecemeal fashion through an anterior approach. Inter-body fusion C5–T1 completed the procedure. During a follow-up period of 12 weeks her paraparesis had recovered almost completely.[10] Naidu *et al.* reported an intraosseous schwannoma involving the bodies of the third and fourth cervical vertebrae causing tetraparesis. This patient was also diagnosed with skeletal fluorosis. The tumour was resected *in toto* and the patient recovered completely.[3]

Intraosseous schwannoma has a clearly recognisable capsule, as we saw in our case. As the tumour grows it perforates the cortex presumably through a natural orifice like a nutrient foramen which then slowly enlarges. In spinal locations neurological compression symptoms will develop when the protrusion is in the direction of the spinal canal. A pure anterior protrusion as in the presented case will cause pain due to spinal instability and to symptoms associated with compression of local structures.

According to the criteria defined by Enneking for the treatment of benign tumours of bone, the treatment of choice is marginal resection.[11] Inadequate surgery of sacral intra-osseous schwannoma is associated with a high recurrence rates.[4,12] However, in other locations adequate curettage has resulted in long-term relief.[1,2,4,13] In general, resection of spinal tumours with marginal margins is difficult to obtain.

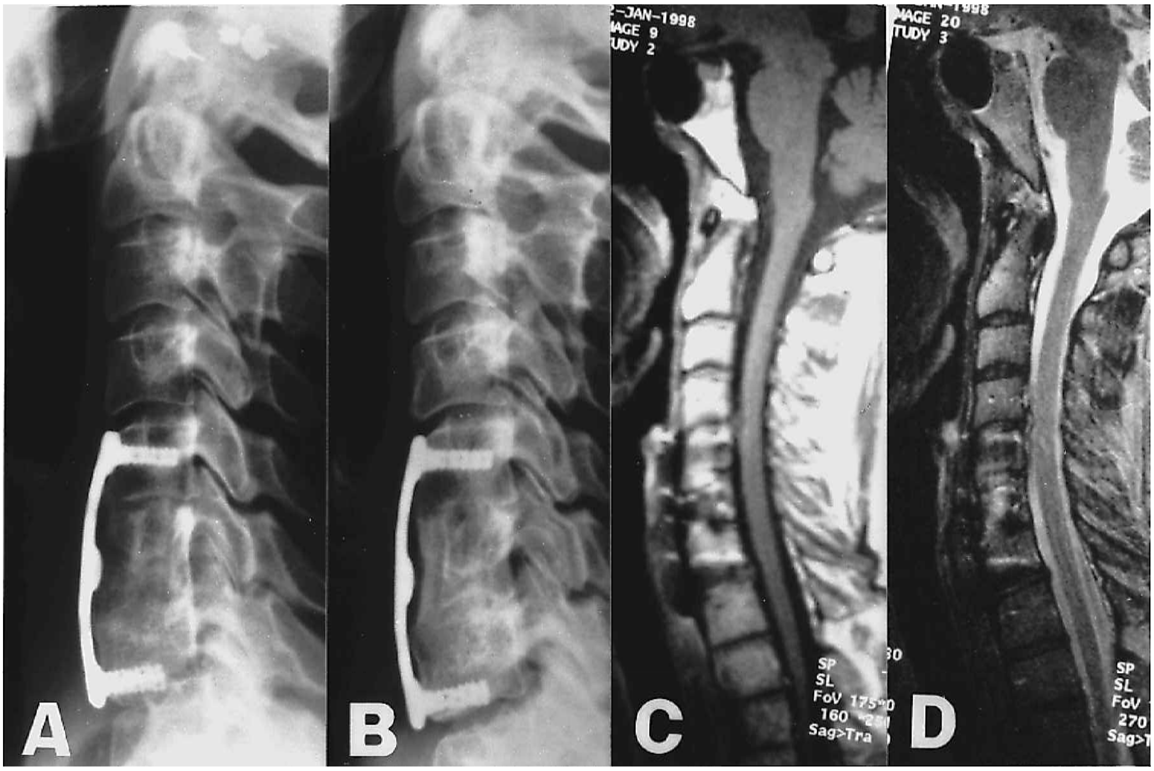

**Fig. 2.** *Six months after curettage and inter-body fusion C5–C7 with tricortical bone plug stabilised with low-profile anterior plate and screws (A) and complete consolidation at 1 year (B). No evidence of local recurrence at 3 years follow-up on MRI (C,D).*

In view of the biological behavior of this particular tumour, accurate intralesional resection (curettage) beyond the original margins of the tumour without local adjuvant therapy seems to be sufficient, as is in our case with a follow-up of 4 years.

Prior to definite surgery of skeletal bone tumours, histological conformation of the presumed diagnosis is mandatory. Biopsy of tumours located in the cervical spine done in a non-contaminating fashion without some degree of exposure is difficult. Therefore we chose intra-operative frozen sectioning after a sufficient exposure to confirm the radiologically *benign* nature of the lesion histologically first, and secondly, when possible, an exact diagnosis of the lesion before definite treatment in the same operation.

Intraosseous schwannoma of the spine is extremely rare. The lesion should be worked-up and dealt with like any other primary skeletal tumour. Intralesional resection by means of thorough curettage seems to be a sufficient treatment.

### References

1 De La Monte SM, Dorfman HD, Chandra R, Malawer M. Intraosseous schwannoma; histologic features, ultrastructure, and review of the literature. *Hum Pathol* 1984; 15(6):551–8.

2 Gordon EJ. Solitary intraosseous neurilemmoma of the tibia. Review of intraosseous neurilemmoma and neurofibroma. *Clin Orthop* 1976; 117:271–82.

3 Naidu MR, Dinakar I, Rao K, Ratnakar KS. Intraosseous schwannoma of the cervical spine associated with skeletal fluorosis. *Clin Neurol Neurosurg* 1988; 90(3):257–60.

4 Turk PS, Peters N, Libbey NP, Wanebo HJ. Diagnosis and management of giant intrasacral schwannoma. *Cancer* 1992; 70(11):2650–7.

5 Ellis GL, Abrams AM, Melrose RJ. Intraosseous benign neural sheath neoplasms of the jaws. Report of seven new cases and review of the literature. *Oral Surg Oral Med Oral Pathol* 1977; 44(5):731–43.

6 Sanado L, Ruiz JL, Laidler L, Polo M. Femoral intraosseous neurilemmoma. *Arch Orthop Trauma Surg* 1991; 110(4):212–3.

7 Agha FP, Lilienfeld RM. Roentgen features of osseous neurilemmoma. *Radiology* 1972; 102(2):325–6.

8 Dickson JH, Waltz TA, Fechner RE. Intraosseous neurilemmoma of the third lumbar vertebra. *J Bone Joint Surg (Am)* 1971; 53(2):349–55.

9 Nooraie H, Taghipour M, Arasteh MM, Daneshbod K, Erfanie MA. Intraosseous schwannoma of T12 with burst fracture of L1. *Arch Orthop Trauma Surg* 1997; 116(6):440–2.

10 Polkey CE. Intraosseous neurilemmoma of the cervical spine causing paraparesis and treated by resection and grafting. *J Neurol Neurosurg Psychiatry* 1975; 38(8):776–81.

11 Enneking WF. A staging system of staging musculoskeletal neoplasms. *Clin Orthop* 1986; 204:9–24.

12 Abernathy CD, Onofrio BM, Scheithauer B, Pairolero PC, Shivis TC. Surgical management of giant sacral schwannomas. *J Neurosurg* 1986; 65(3):286–95.

13 Fawcett KJ, Dahlin DC. Neurilemmoma of bone. *Am J Clin Pathol* 1967; 47(6):759–66.
